# Development of a simple screening method for analyzing cereulide toxin in fried rice using liquid chromatography-tandem mass spectrometry

**DOI:** 10.1007/s11419-024-00683-3

**Published:** 2024-03-22

**Authors:** Hiroshi Koike, Maki Kanda, Chie Monma, Souichi Yoshikawa, Hiroshi Hayashi, Yoko Matsushima, Yumi Ohba, Momoka Hayashi, Natsumi Furuta, Wakaba Okada, Chieko Nagano, Keiko Yokoyama, Tomoko Yokoyama, Takeo Sasamoto

**Affiliations:** 1 Department of Food Safety, Tokyo Metropolitan Institute of Public Health, Tokyo, Japan; 2 Department of Microbiology, Tokyo Metropolitan Institute of Public Health, 3-24-1, Hyakunin-Cho, Shinjuku-Ku, Tokyo, 169-0073 Japan

**Keywords:** Cereulide, Fried rice, Liquid chromatography-tandem mass spectrometry, QuEChERS, Screening

## Abstract

**Purpose:**

The presence of cereulide, an emetic toxin produced by *Bacillus cereus*, in fried rice samples is critical evidence of food poisoning even in situations where *B. cereus* could not be detected. This study aims to develop a screening method for analyzing cereulide in fried rice using the QuEChERS procedure and liquid chromatography-tandem mass spectrometry (LC–MS/MS).

**Methods:**

Cereulide was identified and quantified in fried rice samples using the QuEChERS extraction method and LC–MS/MS. The accuracies of the methods were determined by analyzing fortified blank samples at two concentrations (10 and 50 µg/kg) conducted on three samples daily for five days.

**Results:**

The QuEChERS procedure removed matrix compounds from fried rice. Characteristic MS/MS spectra enabled the identification of cereulide. As the matrix effects in seven fried rice samples were within ± 6%, an external solvent calibration curve could be used for quantification. This method exhibited good accuracy ranging from 88 to 89%. The relative standard deviations for both repeatability and intra-laboratory reproducibility were < 4%. These standard deviations satisfied the criteria of the Japanese validation guidelines for residues (MHLW 2010, Director Notice, Syoku-An No. 1224–1). The limit of quantification was 2 μg/kg. The applicability of this method was confirmed using the analysis of cereulide in fried rice samples incubated with emetic *Bacillus cereus*.

**Conclusions:**

The QuEChERS extraction procedure described herein showed substantial promise as a reliable screening tool for cereulide in fried rice sample.

## Introduction

Cereulide is an emetic toxin produced by subgroups of *Bacillus cereus*, which is widely present in the human environment and can trigger food poisoning and infectious diseases [1, 2]. At least 18 variants of cereulide are produced by emetic *B. cereus* [3]. The cereulide toxin is dodecadepsipeptide [_D_-*O*-Leu-_D_-Ala-_L_-*O*-Val-_L_-Val]_3_, with a molecular mass of 1153 Da [1, 2]. This cyclic peptide is synthesized by non-ribosomal peptide synthetases encoded by the *ces* (cereulide synthetase) gene cluster [3]. Its structural properties make it resilient against digestive enzymes, severe pH conditions, and cooking heat conditions, such as 126 °C for 90 min [1, 4]. Given its stability, cereulide remains active following oral consumption. Therefore, contamination of cooked rice dishes, such as fried rice, pilaf, and pan-fried noodles, is often the cause of outbreaks of cereulide-induced food poisoning [5–7]. Fried rice is the most typical leftover in emetic *B. cereus*-associated food poisoning in Austria and Japan [7, 8]. Since fried rice is a blend of cooked rice and other components, such as eggs, vegetables, meats, and plant oil, differentiating cereulide from complex matrices is challenging. Analyzing cereulide in fried rice provides dependable evidence for the bacterial toxin in suspected foodstuffs.

Traditionally, the detection of viable bacteria and genes has been regarded as important for determining the cause of bacterial food poisoning at an early stage. However, the detection of cereulide is essential for emetic *B. cereus*-associated food poisoning because cereulide can induce nausea and vomiting even if *B. cereus* is dead. Toxic effects can be intense, potentially leading to lethal outcomes [9–11], and the minimum illness-inducing dose in humans is approximately 1 µg [1, 12]. In previous poisoning cases, cereulide has been reported to be the toxin in serum [10] and vomitus sample [13]. Unfortunately, detecting trace amounts of the toxin in biological samples is difficult and rare. Thus, the highly sensitive detection method of cereulide in foods without viable bacteria or toxin-producing genes is required for rapid diagnosis of emetic *B. cereus*-associated food poisoning.

The three primary methodologies used to detect cereulide encompass bioassays, matrix-assisted laser desorption/ionization-time-of-flight mass spectrometry (MALDI-TOF MS), and liquid chromatography–tandem mass spectrometry (LC–MS/MS). The bioassays, such as cytotoxicity assays based on boar sperm mobility [14] and mitochondrial toxicity tests using cell cultures [15–18], have been employed for the detection of toxins. Although those bioassays can detect total toxin activity, they cannot identify the actual toxin causing gastrointestinal illness. Moreover, bioassays suffer from interference from similar molecules, resulting in non-specificity. Recently, MALDI-TOF MS has been widely used in microbiological laboratories [19, 20]. Both bacteria and the cereulide toxin are expected to be detected using MALDI-TOF MS. However, it is challenging to obtain an adequate sensitivity of cereulide because cereulide is a small compound compared to bacteria. Furthermore, the MALDI efficiency of the cereulide has not been adjusted for each sample. If food-derived compounds suppress and decrease the MALDI efficiency of cereulide, false-negative results can easily occur. The use of LC–MS/MS is considered the standard method for cereulide quantification [5, 6, 13, 21–24]. The analysis of cereulide in a typical leftover (fried rice) demands applying a sample preparation method that isolates the analyzed toxin from the matrices. Most approaches involve liquid–liquid or solid–phase extraction. A heavily-labelled cereulide (^13^C_6_-cereulide) and valinomycin, which has a structure similar to that of cereulide, have been utilized as the internal standard (IS) to quantify cereulide accurately. A previous study [6] revealed that ^13^C_6_-cereulide outperforms validamycin as an IS for accurate quantification, but producing ^13^C_6_-cereulide involves a customized peptide synthesis service and is expensive, limiting the suitability of this method for laboratories. Therefore, an easy, simple, and robust method for quantifying cereulide content in fried rice is needed in research laboratories.

Based on prior information on food poisoning, laboratories may speculate on the presence of pathogenic bacteria or the concentration of cereulide in leftovers. To minimize accidental and secondary contamination on the surfaces of the containers and instruments, laboratories and forensic toxicologists need a safer sample preparation method. In 2003, Anastassiades et al. [25] developed a new sample preparation technique, “QuEChERS” (a portmanteau word formed from “quick, easy, cheap, effective, rugged, and safe”). The original technique combines two extraction processes: classical sample extraction (liquid–liquid or liquid–solid extraction) and extract purification via a dispersive solid-phase extraction (d-SPE) process using different sorbents. Due to its simplicity, high flexibility, low solvent usage, and minimization of waste, the technique has been continuously modified to analyze drugs and toxins [26–29]. The modified QuEChERS has become popular as a sample preparation method in analytical procedures for an ever-increasing number of compounds in different matrices. A previous study [8] utilized QuEChERS extraction for cereulide with d-SPE as a clean-up step and quantified cereulide in fried rice with the heavily-labelled IS. In the event of a large-scale food poisoning, it may be necessary to test countless samples, which may not be possible using special tools. To overcome the problems, a more time-saving and cost-effective method is needed.

In this study, we developed to determine the concentration of cereulide in fried rice employing a QuEChERS method using LC–MS/MS without IS. Furthermore, we evaluated the applicability and practicality of the method by analyzing cereulide in fried rice sample spiked with emetic *B. cereus*.

## Materials and methods

### Reagents and materials

A synthetic cereulide peptide standard solution (50 µg/mL dissolved in methanol) was procured from Fujifilm Wako Pure Chemical Corp. (Osaka, Japan). When handling this toxin, appropriate precautions, including the use of gloves and personal protective equipment, should be followed. Surfaces and materials that encountered the toxin need to be cleaned with bleach to eliminate any residual toxins.

Acetonitrile (HPLC grade), ammonium formate (LC/MS grade), formic acid (LC/MS grade), magnesium sulfate (analytical grade), methanol (HPLC grade), sodium chloride (analytical grade), and dehydrated trisodium citrate (analytical grade) were also procured from Fujifilm Wako Pure Chemical Corp. Ultrapure water (resistance > 18 MΩ) was sourced through a Milli-Q water system (Merck, Billerica, USA). Brain heart infusion (BHI) broth and mannitol egg yolk polymyxin (MYP) agar plates were procured from Nissui Pharmaceutical Corp. Ltd. (Tokyo, Japan).

The Stomacher-80 T was obtained from Organo Co. (Tokyo, Japan). An individual polyethylene sterile stomacher bag, size 100 × 150 × 0.09 mm, with a capacity of 80 mL was purchased from Organo Co. The 0.45 µm membrane filter, Millex HV 0.45 µm, was purchased from Merck.

Seven different cereulide-free fried rice samples were purchased from local supermarkets in Tokyo to use the method’s optimization and validation, and to check selectivity. All samples were entirely free from innate cereulide. One fried rice sample (pilaf) was confirmed to be free of *B. cereus*.

### Standard solutions

A stock standard solution was prepared by diluting the original standard solution with acetonitrile to achieve a concentration of 5 µg/mL. The solution was stored at 4 °C until it was required for analysis and remained stable for one year. A working standard solution was prepared by dilution with acetonitrile to a final concentration of 1 µg/mL. The diluted solutions were stored at 4 °C for up to one week.

### LC–MS/MS conditions

The LC–MS/MS conditions were consistent with those outlined in a previous study [30]. In summary, LC separations were carried out using an ACQUITY UPLC H-Class system (Waters Co., Milford, MA) fitted with a YMC Triart C18 column (2.1 mm i.d. × 100 mm, 3 µm, YMC Co. Ltd, Kyoto, Japan), which was kept at 45 °C. A gradient was applied using 1 mmol/L ammonium formate in water containing 0.1% formic acid (A) and 0.1% formic acid in methanol (B) as the mobile phase. The flow rate and injection volume were set at 0.3 mL/min and 2 µL, respectively. Mass spectrometry was carried out using a QTRAP 5500 instrument (Sciex, MA, USA). The mass detector was operated according to the parameters listed in Table 1. The MS resolution value was defined to correspond to 0.7 Da. For searching MS parameters, “the suitable adduct ion” was optimized in the range of *m/z* 1100–1200, and the “product ion spectra” were expanded in the range of *m/z* 100–1200 by steps of *m/z* 100. The multiple reaction monitoring (MRM) information-dependent acquisition (IDA) parameters incorporated the acquisition of one ion with a peak height exceeding 1000 counts/s and a step size of 0.12 Da. Both the MRM transitions and product ion scanning were carried out within one cycle.

**Table 1:** Summary of MS/MS transitions and parameters for cereulide

Retention time (min)	Precursor ion (*m/z*)	Production (*m/z*)	Declustering potential (V)	Collision energy (eV)	Collision cell exit potential (V)	Ion ratio^a^ (%)		
						Standard^b^	Fortified sample^c^	*B. cereus* spiked samples^d^
19.7	1170.7	1125.7^e^	96	51	8	–	–	–
		499.4		73	10	66.6	67.7	61.5
		385.2		75	16	50.6	51.3	48.7
		357.2		83	24	84.0	86.2	83.4
		314.2		85	10	67.5	71.3	64.4
		172.2		117	10	56.0	55.8	55.3

^a^The relative ion abundance ratio of each product ion

^b^In the standard solution (40 µg/kg)

^c^In the sample fortified with 50 µg/kg

^d^In the *B. cereus* spiked sample

^e^Quantification ion

### Sample preparation

The sample preparation used for method optimization and validation was performed in a conventional laboratory (P1 level). The QuEChERS procedure was applied in the following steps: an exact 5.0 g sample of fried rice was transferred into a stomacher bag and combined with 2.5 mL of water. The mixture was homogenized using a Stomacher-80 T for one minute at a standard speed. To minimize bacterial contamination, the homogenization method was optimized by comparing the extraction technique using a homogenizer and a stomacher bag. Subsequently, acetonitrile (15 mL), containing 0.5% formic acid, was incorporated into the mixture. Following another round of homogenization, the mixture was transferred to a new 50 mL polypropylene centrifuge tube. This was followed by the addition of 4 g of magnesium sulfate, 1.5 g of trisodium citrate dihydrate, and 1 g of sodium chloride. This mixture was then immediately shaken manually for a minute. The mixture was centrifuged at 1,840 × *g* for 10 min at 4 °C. The supernatant was transferred into a 20 mL volumetric flask and brought up to a volume of 20 mL with acetonitrile containing 0.5% formic acid. A portion (1 mL) of the examination solution was transferred to a 1.5 mL microtube and centrifuged at 13,400 × *g* for 5 min at 4 °C. The supernatant was subsequently transferred to a glass vial (the toxin was calculated to be diluted four-﻿fold with this sample preparation).

### Calibration curves

Quantification values were derived from the external solvent calibration curves without weighting. Standard solutions used to develop the calibration curves were prepared using acetonitrile containing 0.5% formic acid, and appropriate volumes of the working solution were added to achieve the final concentrations of 0.5, 1, 2.5, 5, 10, and 25 µg/L. As a result, the concentration of cereulide in fried rice can be quantified as 2, 4, 10, 20, 40, and 100 µg/kg.

### Single-laboratory method validation

The accuracy of the devised method was evaluated based on the Guidelines for the Validation of Analytical Methods for Residual Agricultural Chemicals in Food established by the Ministry of Health, Labour, and Welfare of Japan [31]. The recovery (trueness) in this study was defined as a percentage using the formula:

$$Recovery \left( {trueness, \% } \right) = \left( {Measured\; concentration/Theoritical\; concentration} \right) \times 100.$$

The trueness, relative standard deviation of repeatability (RSD_r_), and within-laboratory reproducibility (RSD_WR_) were ascertained by conducting fortified recovery tests at two different concentrations (10 and 50 µg/kg), using three samples daily over five distinct days. A 50 or 250 µL aliquot of the working standard solution was added to 5.0 g of the samples, left standing for 30 min before extraction, and the results were analyzed by one-way analysis of variance (ANOVA). Selectivity was ascertained by analyzing blank samples. The limit of quantification (LOQ) was gauged by adding cereulide to blank samples to achieve final concentrations of 2, 4, and 8 µg/kg. These values were considered as the lowest sample concentrations yielding a signal-to-noise (S/N) ratio exceeding 10. The limit of detection (LOD) was defined as the lowest concentration with cereulide present and an S/N ratio of at least 3. The relative expanded measurement uncertainty (U) was estimated according to the Eurachem/CITAC guide [32]. The U percentage was calculated as the ratio of the coverage factor (k = 2) to the RSD_WR_ using the equation:

$$U\left( \% \right) = k \times RSD_{WR}.$$

The matrix effect (ME) was assessed by comparing the ratios of each slope between the external solvent calibration curves (Se) and the matrix-matched calibration curves (Sm) across six concentration levels (2, 4, 10, 20, 40, and 100 µg/kg). The ME was computed as a percentage using the formula:

$$ME\left( \% \right) = \left( {Sm/Se - 1} \right) \times 100.$$

Matrix-matched calibration standard solutions were generated by combining an aliquot of extracted blank solution (900 µL) and an appropriate concentration of the standard solution (100 µL) used for the calibration curves.

### Application to positive sample

All bacterial experiments were conducted in a biosafety level 2 (BSL-2) laboratory. To simulate the presence of cereulide in leftovers, a fried rice sample (pilaf) was incubated with emetic *B. cereus*. The fried rice sample was spiked with the emetic *B. cereus* BC 90–37 and incubated to produce cereulide. *B. cereus* BC 90–37, identified as a cereulide-producing strain, was isolated from the causative pilaf of an emetic *B. cereus*-associated food poisoning in Tokyo, Japan. Initially, the BHI broth culture of *B. cereus* BC90-37 was adjusted to 1.0 × 10^3^ cfu/g. After incubation, BHI was combined with 50.0 g of fried rice (three replicates). The mixture was then inoculated with 1.0 × 10^3^ cfu/g *B. cereus* BC 90–37 and incubated at 37 °C for 16 h. After incubation, a 5.0 g aliquot was taken from each sample. They were analyzed within one hour after weighing. The *B. cereus* spiked sample was prepared as aforementioned. After centrifugation at 13,400 × g for 5 min at 4 °C, a part (0.8 mL) of the supernatant was further sterilized by filtration a 0.45 µm membrane filter, allowing cereulide to pass through while removing emetic *B. cereus*. The elute was transferred to a glass vial. The vial of the *B. cereus* spiked sample was then outside the BSL-2 laboratory. To investigate whether cereulide adsorbs on the membrane filter, a fortified test was executed at a concentration of 50 µg/kg cereulide (three replicates) as an internal quality control (QC). The QC and *B. cereus* spiked sample were prepared in the same batch.

## Results

### Optimization

#### LC–MS/MS conditions

**Fig. 1:**
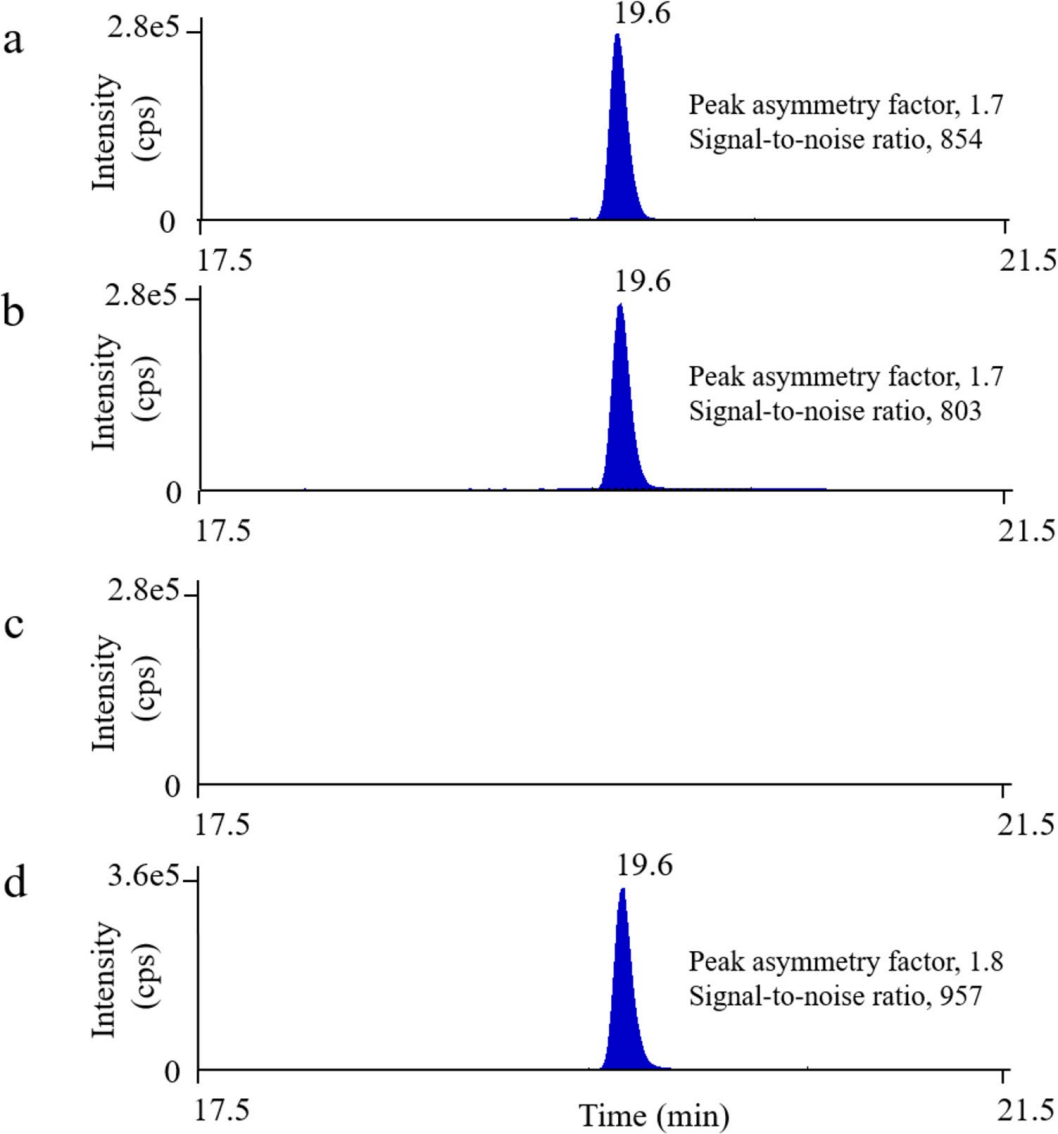
LC–MS/MS chromatograms, *m/z* 1170.7 → 1125.7, of **a** cereulide standard solution (40 µg/kg), **b** matrix standard solution (40 µg/kg), **c** blank sample, and **d** *B. cereus* spiked samples (sample No.3; see Table 3)

Cereulide is known for its high affinity for ammonium ions [22], and the ammonium adduct [M + NH_4_]^+^, with a molecular mass of *m/z* 1170.7, was found to be the most abundant in this study. To reliably identify cereulide in fried rice, MS/MS scans were performed. The six characteristic product ions listed in Table 1 were observed by the MS/MS results, as reported by a previous study [33]. When the relative ion ratios of the fried rice sample fortified with cereulide were compared with those of the standard shown in Table 1, the relative ion ratios were kept within limits (± 20%). The results satisfied the identification criteria in several guidelines [34, 35]. Figure 1 shows the LC–MS/MS chromatograms obtained from (a) the cereulide standard solution (40 µg/kg) and (b) the matrix standard solution (40 µg/kg). No peak tailing or leading was observed in the presence of matrix compounds. The background noise did not increase significantly.

**Fig. 2:**
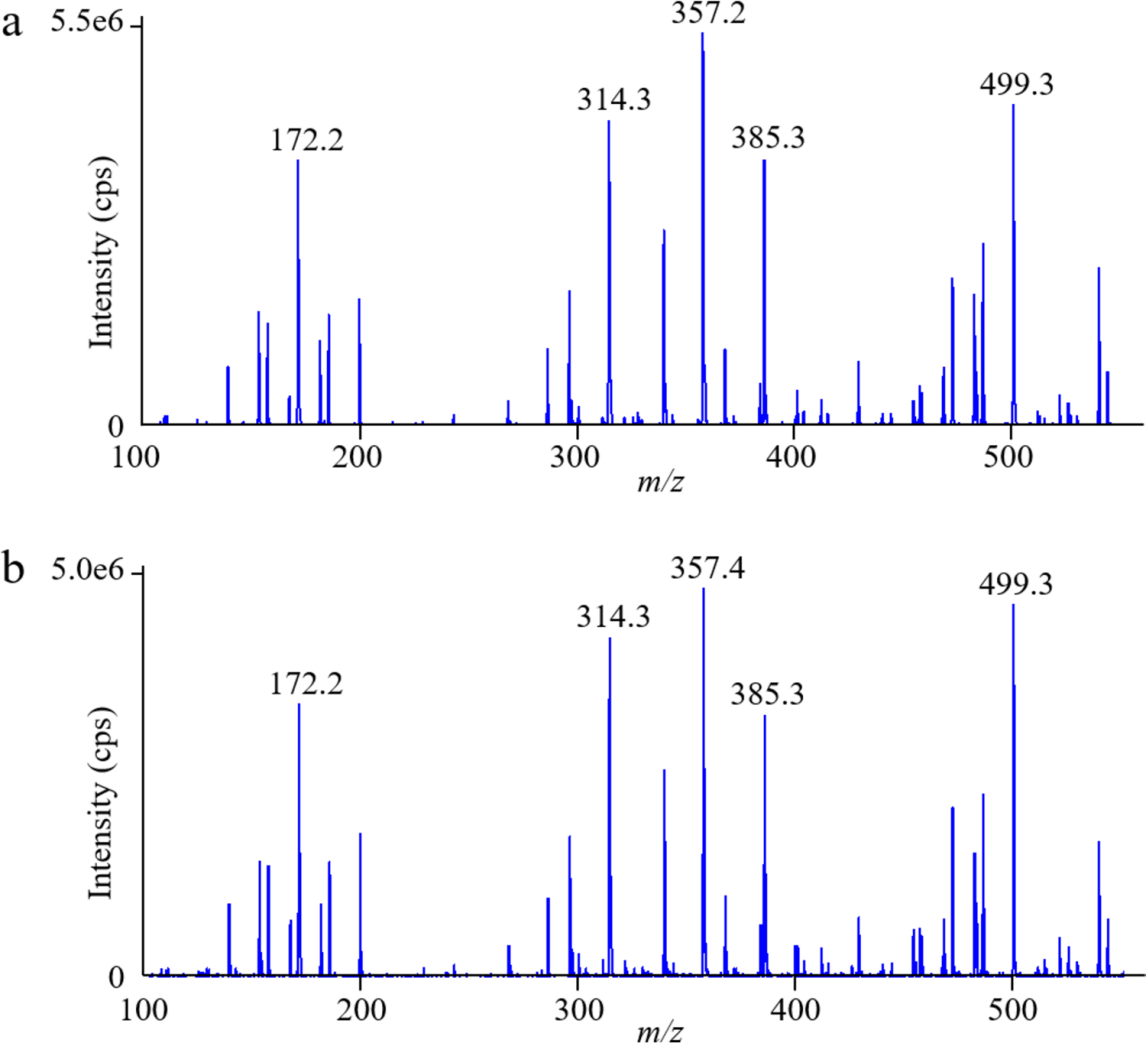
Results of the product ion spectra analysis of *m/z* 1170.7 in **a** fried rice sample fortified with cereulide as 50 µg/kg and **b** *B. cereus* spiked samples

Figure 2 (a) shows typical product ion spectra in the fried rice sample fortified with 50 µg/kg cereulide. Given the balance between the characteristic structure and sensitivity of cereulide fragments, we narrowed down the MS/MS spectra range from *m/z* 100 to 550. When compared using a library search, the target MS/MS spectra of the fortified sample and the standard yielded a purity fit over 99%. The results corresponded to the cutoff value (> 70%) for identification in routine use [36, 37].

#### Extraction procedure

The sample preparation procedures were based on the previous study [38], and homogenization by using homogenizer was initially adopted as an extraction procedure. In the fortified tests using fried rice samples with 10 µg/kg cereulide (*n* = 3), the recovery and RSD were 83.4% and 0.3%, respectively. However, preventing bacterial contamination during sample preparation is crucial for identifying the cause of bacterial food poisoning. Residual bacteria can adhere to a homogenizer and contaminate subsequent runs, necessitating the independent homogenization of each sample in a confined space. Homogenization using stomacher bags was investigated to address this issue. When stomacher bags were used, the recovery and RSD were 87.3% and 1.7%, respectively. The extraction efficiency of cereulide did not significantly decrease. Therefore, the stomacher bag was used in this study.

### Single-laboratory method validation

#### Linearity and ME

Linearity was evaluated by external solvent calibrations in the range of 2–100 µg/kg.　A typical calibration equation is as follows:

$$y = 41,468 x + 27,571,$$

where x is the concentration (µg/kg) and y is the peak absolute area. The calibration curve showed good linearity with the coefficient of determination (R^2^) over 0.999. At all calibration points, the relative ion ratios listed in Table 1 remained constant.

We adopted high-speed centrifugation as the clean-up step. To evaluate the influence of the electrospray ionization efficiency, ME was calculated in seven different fried rice. The MEs of cereulide ranged from -2.2% to 5.8%. The ME values were acceptable according to the criteria (± 20%) outlined in the guideline [34].

#### Trueness and precision

The accuracy of this method was evaluated by calculating the trueness and precision through fortified recovery tests and the results are summarized in Table 2. The results satisfied the criteria set by the Japanese guidelines [31], which demand recovery rates of 70%–120%, an RSD_r_ of < 25%, and an RSD_WR_ of < 30% at a fortified level of 10 µg/kg. The estimated U was acceptable (i.e., ≤ 50%) [34].

**Table 2:** Validation results for cereulide in fried rice

Fortification level (µg/kg)	Trueness (%)	RSD_r_^a^ (%)	RSD_WR_^b^ (%)	LOQ (µg/kg)	Uncertainty^c^ (%)
10	88.5	3.4	3.4	2	8.7
50	87.6	3.7	4.4		

^a^RSD of repeatability

^b^RSD of within-laboratory reproducibility

^c^The relative expanded measurement uncertainty with a coverage factor (*k* = 2)

#### Selectivity, LOQ, and LOD

Selectivity was confirmed by analyzing seven blank samples. Figure 1 (c) displays the typical LC–MS/MS chromatograms of a blank sample (*m/z* 1170.7 → 1125.7). No interfering peaks were seen at the same retention time as cereulide. The LOQ was set to 2 µg/kg, and the estimated LOD was 0.7 µg/kg. To reassess our method using fortified recovery tests at the LOQ level in fried rice (*n* = 3), the recovery and RSD_r_ were 80.2% and 7.4%, respectively. Using MRM-IDA experiments, the MS/MS spectra (*m/z* 1170.7 → 100–550) were sufficient at the LOQ level.

### Application to positive sample

Fried rice was inoculated with emetic *B. cereus t*o confirm the suitability of the method for identifying and quantifying cereulide in positive samples. Our method determined the cereulide produced by emetic *B. cereus* (Fig. 1 (d)), and the relative ion ratios matched those of the standard solutions (Table 1). Moreover, in the analysis of the product ion spectra focusing on *m/z* 1170.7 (Fig. 2(b)), the purity fit value was 99%. These findings conclusively show that product ion scanning can identify cereulide produced by emetic *B. cereus* in fried rice. After the filter sterilization process, the cereulide concentration of the elute exceeded the range of the calibration curves. Then, the elute was diluted ten-fold with acetonitrile containing 0.5% formic acid (the *B. cereus* spiked sample was diluted 40-fold in sample preparation).

The results are shown in Table 3. The found value and RSD of cereulide were 519 μg/kg and 17.7%, respectively. The count and RSD of *B. cereus* were 1.9 × 10^8^ cfu/g and 27.2%, respectively. This variation is likely due to differences in growing bacterial counts. The biosafety regulation requires the filter sterilization of the *B. cereus* spiked sample. As the QC results, the average recovery and RSD were 80.1% and 0.3%, respectively. Large losses or strong adsorption were not observed. The satisfactory result on the QC confirmed the accuracy of the analysis of positive samples.

**Table 3:** Comparison of *Bacillus cereus* count and cereulide content in fried rice after incubation

No.	Count of *B. cereus* (cfu/g)	Content of cereulide (µg/kg)
1	1.2 × 10^8^	417.6
2	2.4 × 10^8^	639.9
3	2.2 × 10^8^	499.6
Average, RSD^a^	1.9 × 10^8^, 27.2	519.0, 17.7

^a^Relative standard deviation (%)

## Discussion

A sample clean-up process is required for reliable identification of cereulide in fried rice samples. Previous reports [8, 39] required clean-up procedures utilizing d-SPE and defatting with *n*-hexane. Since this procedure is time-consuming (1–2 h), we considered that to be a bottleneck in the response to a large-scale poisoning incident. We consumed less time and achieved simple purification. Owing to these practical benefits, the developed approach provides evidence for rapid diagnosis of emetic *B. cereus-*associated food poisoning.

A calibration curve with the heavily labelled IS is one of the most scientific techniques for accurate quantification. The IS method has advantages for correcting human and operational errors. However, it is not realistic to handle countless samples using the IS method. Considering the difficulties, we proposed a simple screening method with an external solvent calibration curve. We are conducting further research to analyze cereulide in suspected foodstuffs.

Parameters such as LOD and LOQ play a key role in comparing the intake to the minimum illness-inducing dose. Fried rice, one of the most popular leftover foods in Japan, is often associated with emetic B. cereus-induced food poisoning [7]. The consumption of fried rice in Japan is estimated at approximately 400 g per capita per meal. Based on the lowest cereulide dose known to cause illness (1 μg), the LOQ in this study could be monitored to detect cereulide that potentially causes illness.

## Conclusion

The validity and applicability of the optimized method demonstrate the practicality of the QuEChERS extraction procedure for cereulide. Owing to the absence of d-SPE clean-up and the use of an IS, this new method provides ease of determination of this toxin in fried rice. The simplicity reduced the risk of bacterial contamination in laboratory instruments. The product ion spectra enabled us to distinctly identify the presence of cereulide in fried rice without false-negative or false-positive outcomes. Furthermore, the validated method successfully quantified cereulide in *B. cereus* spiked samples. The developed method is expected to be applicable to actual leftovers and biological samples of food poisoning.

## Data Availability

The datasets generated and/or analyzed during the current study are available from the corresponding author upon reasonable request.
